# A Review in Research Progress Concerning m6A Methylation and Immunoregulation

**DOI:** 10.3389/fimmu.2019.00922

**Published:** 2019-04-26

**Authors:** Caiyan Zhang, Jinrong Fu, Yufeng Zhou

**Affiliations:** ^1^Children's Hospital and Institutes of Biomedical Sciences, Fudan University, Shanghai, China; ^2^NHC Key Laboratory of Neonatal Diseases, Fudan University, Shanghai, China

**Keywords:** N6-methyladenosine, binding proteins, mechanisms, immunoregulation, cellular functions

## Abstract

Over 100 types of cellular RNA modifications have been identified in both coding and a variety of non-coding RNAs. N6-methyladenosine (m6A) is the most prevalent and abundant post-transcriptional RNA modification on eukaryote mRNA, and its biological functions are mediated by special binding proteins (i.e., methyltransferases, demethylases, and effectors) that recognize this modification. The presence of m6A on transcripts contributes to diverse fundamental cellular functions, such as pre-mRNA splicing, nuclear transport, stability, translation, and microRNA biogenesis, implying an association with numerous human diseases. This review principally summarizes recent progress in the study of m6A methylation mechanisms and relevant roles they play in immunoregulation.

## Introduction

In 1970, m6A was first recognized as an abundant nucleotide modification in eukaryotic messenger RNA (1), by far the most prevalently known of over 100 kinds of RNA modifications identified in various classes of RNAs, including mRNA, rRNA, tRNA, snRNA, microRNA (miRNA), and long non-coding RNA (lncRNA) (2). m6A is present in 0.1–0.4% of all adenosines in global cellular RNAs and accounts for ~50% of all methylated ribonucleotides (3). However, little was known about its potential functional significance and extent transcript identities until very recently. m6A occurs primarily in two consensus sequence motifs, G m6A C (~70%) and A m6A C (~30%) (4, 5). Long internal exons, locations upstream of stop codons, and the 3′-UTR of mRNA are preferred modification sites for m6A, implying roles involving translational control, influencing affinities of RNA binding proteins or unique m6A-derived transcriptome topology (6–9).

The discovery of proteins involved in m6A regulation has been among the most significant achievements in this area of study, elucidating their roles as “writers” (m6A methyltransferases), “erasers” (m6A demethyltransferases), and “readers” (effectors recognizing m6A) (10). Methyltransferase like-3 (METTL3, also known as MT-A70) was among the first of all identified core writer components, responsible for installing m6A on RNA (11), Other core enzyme components such as METTL14 (12, 13), in addition to accessory components including Wilms′ Tumor 1-associating Protein (WTAP) (12, 14), KIAA1429 (15), RNA binding motif protein 15 (RBM15), RBM15 paralog (RBM15B) (16), and zinc finger protein 217 (ZFP217) (17) have also been studied. The METTL3 complex acts at the consensus RRACH motif (R = A or G, H = A, C, or U) (18). METTL3 or METTL14 depletion reduces the ratio of m6A/A, while knockdown of WTAP decreases amounts of METTL3 complex bound to RNA, implying that WTAP may be responsible for the recruitment of RNAs (12, 14). m6A demethylase fat mass and obesity-associated (FTO) protein was the first recognized “eraser” enzyme that reverses RNA modification and controls cellular homeostasis (19). ALKB homolog 5 (ALKBH5) was also recognized as a demethylase involved in alkylated DNA repair (20). Three “reader” proteins that can directly interact with m6A sites via their YTH domains have been discovered to date. YTH domain-containing family (YTHDF) proteins 1 and 3 promote translation of m6A-modified mRNA via interaction with translation initiation factors (21, 22), whereas YTHDF2 promotes RNA degradation via recruitment of m6A modified mRNA to nuclear processing bodies (P bodies) (23).

**Figure 1:**
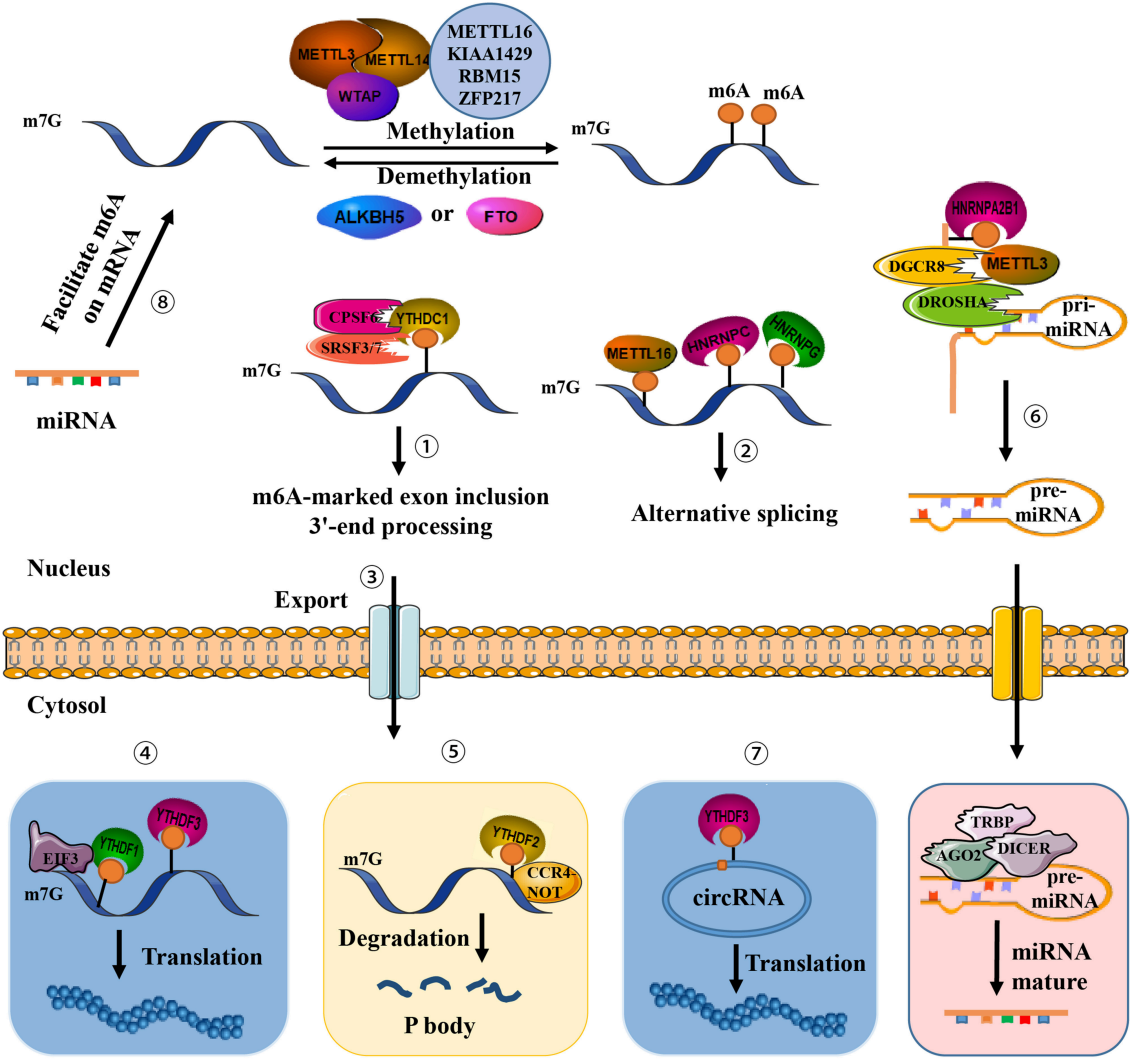
Presence of m6A on transcripts contributes to diverse fundamental cellular functions. ①-⑤ m6A regulates exon inclusion and 3′-end processing, alternative splicing, nuclear export, translation, and degradation. ⑥ m6A regulates miRNA processing. ⑦ m6A regulates circRNA translation. ⑧ miRNA regulates m6A formation.

m6A as an mRNA modification that is abundant in some viruses and nearly all eukaryotes (2). A variety of cytopathologic processes involving nuclear RNA export, splicing, mRNA stability, circRNA translation, miRNA biogenesis, and lncRNA metabolism have recently been linked to aberrant levels of m6A (Figure 1) (24–26). In addition, m6A modification has been associated with numerous physiological and pathological phenomena, including obesity, immunoregulation, yeast meiosis, plant development, and carcinogenesis (2, 27).

This review summarizes the most recent progress in research concerning m6A and analyzes newly identified roles this modification plays in the regulation of gene expression and immune responses.

## m6A Writers, Erasers, and Readers

### Writers

A methyltransferase complex within the nuclear speckle, mainly consisting of METTL3 and METTL14, installs m6A modification on distinct target RNAs via the methyl groups of S-adenosylmethionine (SAM) transferase (11, 12). Two subunits constitute an m6A methyltransferase complex: MT-A (200-kDa) and MT-B (800-kDa). METTLE3 (or MT-A70), a 70-kDa protein, was first identified in 1997 and possesses a SAM-binding domain and a DPPW motif (Asp-Pro-Pro-Trp). It serves as a catalytic subunit and constituent of a 200-kDa methyltransferase complex isolated from the nuclear extract of HeLa cells (11). Knockdown of METTL3 leads to decreased m6A levels and concomitant apoptosis of human HeLa and HepG2 cells (12). The other core writer component, METTL14, harbors two conserved functional domains, a SAM-binding domain and an EPPL motif (Glu-Pro-Pro-Leu) involved in catalyzing the methylation reaction, an additional N-terminal coiled-coil domain for mediating protein–protein interaction, and a G-rich sequence at the C-terminal end (10). METTL3-METTL14, in a ratio of 1:1, form a stable heterodimer (1 MDa) localized at nuclear speckles (28). In addition, METTL14 supports METTL3 in recognizing special RNA substrates. The heterodimer preferentially methylates RNA substrates exhibiting a consensus GGACU domain and contains a moderate preference for substrates with less structure (29). WTAP, a splicing factor, acts as the third crucial component of the writer. Although it does not possess any recognizable domains or motifs, WTAP binds to the METTL3-METTL14 heterodimer and abundantly regulates m6A deposition inside cells (12). WTAP may mediate the position of the heterodimer on nuclear speckles and recruit target RNA for m6A modification (14), thus indirectly enhancing the catalytic capacity of the writer. WTAP may also recruit unknown factors to bind to the methyltransferase complex and modulate methylation.

### Erasers

FTO is the first discovered demethylase that removes methyl groups from m6A (19), indicating that m6A is a dynamically reversible RNA modification. FTO modulates alternative splicing of RUNX1T1, which is involved in adipogenesis (30), as well as the 3′-end mRNA processing in 293T cells (31). ALKBH5 is the second identified demethylase and exhibits distinct physiological functions (20). An ALKB domain is commonly situated in the middle regions of FTO and ALKBH5, consisting of two active motifs termed as HXDXnH and RXXXXXR (X = any amino acid), which binds to Fe(II), as well as α-ketogluterate (α-KG) and substrate, respectively. Compared to ALKBH5, a distinctive fold mediates protein interaction at the C-terminal end of FTO. The N-terminus of ALKBH5 is characterized by an additional A-rich motif responsible for localizing ALKBH5 at nuclear speckles (10). FTO and ALKBH5 have substantial tissue-specificity and diverse intracellular localization. FTO has been reported to be highly abundant in adipose and cerebral tissue, while ALKBH5 has been reported to be primarily expressed in the testes (20). Thus, demethylation in some tissues may be performed solely by either FTO or ALKBH5.

### Readers

While writer proteins install m6A at a specific domain on target RNA, altering its secondary or tertiary structure (32), another class of proteins, termed readers, recognize, and preferentially bind the RNA to confer its fate and regulate downstream functions. An RNA-pull down assay initially revealed that YTH domain-containing family proteins YTHDF1-3 rich in the P/Q/N motif were discovered in mammalian cells to be m6A readers that recognize the consensus sequence G[G > A]m6ACU (7, 23). Among this protein family, YTHDF2 has the strongest affinity to localize P bodies via its P/Q/N motif where the concentration of mRNA turnover factors in facilitating RNA degradation (23). Notably, YTHDF2 recruits the deadenylase complex CCR4-NOT via the YTHDF2 N-terminus and mediates RNA degradation in mammalian cells (33). YTHDF1 was found to interact with translation initiation factors to evoke m6A-containing mRNA translation (21). YTHDC1, a YTH domain-containing protein, was subsequently validated as a nuclear m6A reader and showed almost completely overlapping sites with m6A in nuclear RNAs. YTHDC1 recruits the serine and arginine-rich splicing factor 3 (SRSF3), restricts exon-skipping factor SRSF10 binding, and promotes exon inclusion (34). YTHDC1 also plays a critical role in pre-mRNA processing in the oocyte nucleus via interaction with the pre-mRNA 3′-end processing factors CPSF6, SRSF3, and SRSF7 (35). A nuclear m6A reader has been identified recently within the pre-mRNA consensus motif RRACH that destabilizes the stem structure, enabling the U-tract motif to become exposed as a single-strand and become more accessible for heterogeneous nuclear ribonucleoprotein (hnRNP) HNRNPC binding, altering alternative splicing of target RNA (36). HNRNPG is another critical protein containing a low-complexity domain at its C-terminus. Arg-Gly-Gly repeat sequences bind directly m6A sites and alter the expression and alternative splicing pattern of target mRNA (37). Additionally, the hnRNP family protein HNRNPA2B1 binds to the m6A RGAC motif in a subset of primary miRNA transcripts, recruits a microprocessor complex to facilitate miRNA processing, and elicits alternative splicing effects similar to those of METTL (37, 38).

A number of m6A readers have been recently identified in addition to the aforementioned proteins. FMR1, a protein that contains a RGG domain, three KH domains, and two Agenet domains at its N-terminus, preferentially recognizes the sequence GGm6ACU via RGG domain binding and represses translation by stalling ribosomal translocation (39, 40). Further data revealed that a class of proteins termed m6A-repelled proteins preferentially bind only to unmodified mRNA. The stress granule proteins G3BP1 and G3BP2 are reported to be the most forceful of the repelled proteins. G3BP1 consistently interacts with the GGACU but not the GGm6ACU motif to stabilize target RNA (39, 41). Interestingly, METTL16 serves as a both m6A writer and reader of U6 snRNA, and is concerned with mRNA splicing (42). METTL16-dependent sites are mainly located in introns or exon-intron boundaries, unlike common m6A sites.

## Research Techniques for m6A

### m6A Seq and MeRIP-Seq

The profile of m6A sites throughout the transcriptome remained unclear until two independent sequencing methods, m6A Seq and MeRIP-Seq (m6A-specific methylated RNA immunoprecipitation (IP) with next-generation sequencing), were established in 2012 (7, 9). In brief, the mRNA is first randomly fragmented into approximately 100 nt prior to IP using m6A-specific antibody. Then, RNA-seq is applied to the RNA pool, in which m6A-tagged RNA fragments are enriched. These two methods revealed m6A to be a pervasive and dynamically reversible modification, particularly enriched in 3′-UTR regions and near mRNA stop codons. It was also reported that m6A sites in some RNAs have very high levels of conservation between human and mouse transcriptomes. Although the aforementioned research methods are easily and effectively applied toward the field of epitranscriptomics, a resolution of approximately 200 nt is impractical to precisely identify m6A positions.

### PA-m6A Seq

PA-m6A-seq (photo-crosslinking-assisted m6A sequencing) has recently been developed as one of two UV-induced RNA-antibody crosslinking strategies (43). It significantly improves resolution on the basis of m6A-seq/MeRIP-seq. Addition of 4-thiouridine (4SU) into medium results in it embedding into RNA. This is followed by anti-m6A antibody IP with subsequent m6A-containing RNA crosslinking using 365-nm UV light. Afterwards, RNase T1 digests the crosslinked RNA to about 30 nt, allowing for efficient sequencing. Within approximately 23 nt, single consensus methylation sequences can be determined by PA-m6A-seq at single-base resolution. However, m6A sites cannot be detected if the distance between them and those of 4SU incorporation is too great. This UV crosslinking strategy effectively provides insight into m6A-containing RNA and RNA-binding proteins.

### m6A-CLIP/IP and miCLIP

Additional UV crosslinking strategies are known as m6A-CLIP/IP and miCLIP (m6A individual-nucleotide-resolution crosslinking and immunoprecipitation) (8, 44). Under 254-nm UV light, crosslinking can be brought about between RNA fragments and anti-m6A antibodies. The crosslinked fragments are subsequently retrieved by applying proteinase K and reverse transcription, thereby leading to highly specific mapping of mutation or truncation profiles to precise m6A sites at single-nucleotide resolution throughout the transcriptome.

### SCARLET and m6A-LAIC-seq

To determine the m6A status of a random mRNA or lncRNA from a total RNA pool rather than from purified distinct RNAs and quantify m6A stoichiometry at specific locations, SCARLET (site-specific cleavage and radioactive-labeling followed by ligation-assisted extraction and thin-layer chromatography) was developed in 2013 (45). To achieve site-specific cleavage, RNase H is added to the total RNA sample followed with radiolabeling using ^32^P. Labeled RNA fragments are then splint-ligated to DNA oligonucleotides by DNA ligase. All RNAs are subsequently digested completely with RNases T1/A, but ^32^P-labeled sites remain protected from digestion. Finally, after gel purification and nuclease P1 digestion, samples are analyzed using thin-layer chromatography (TLC). Although SCARLET can precisely determine m6A modification sites at single-nucleotide resolution, it is incapable of use in high throughput screening and is time-consuming as a whole (2).

A recently established m6A-LAIC-seq (m6A-level and isoform-characterization sequencing) method can also quantify m6A presence, even on a transcriptome-wide level (46). First, full-length RNA IP is performed employing excess anti m6A antibody. The sample is next treated with External RNA Controls Consortium (ERCC), and the internal standards are supernatant (m6A-negative fraction) and eluate (m6A-positive fraction). In the two pools, an ERCC-normalized RNA enrichment ratio quantifies m6A modification of each gene, followed by library construction and sequencing. m6A-LAIC-seq was found to indicate that under 50% m6A modification levels exist in a majority of genes, and that 3′-ends of RNA molecules containing m6A would become shorter due to proximal alternative polyadenylation sites.

Identifying precise m6A sites in RNA transcripts is a critical step toward comprehending the biological functions of this modification. Although existing methods have been adopted widely in multiple areas and resulted in some achievements, challenges remain at both single-base and quantitative sequencing levels (47). For example, how to relatively distinguish the m6A levels of different sites in a common transcript, and the establish of new methods getting rid of m6A-special antibody will make perfect sense.

## m6A Modification and ncRNA

Identification of m6A methyltransferases, demethylases, and effectors have revealed that m6A modification is critical throughout the whole RNA life cycle, including pri-mRNA splicing, mRNA nuclear transport, molecular stability, translation, and subcellular localization (48), additionally involved in miRNA biogenesis, lncRNA processing, and circRNA functions. m6A modification is especially abundant in circRNA, and only a single m6A residue is sufficient to drive circRNA translation via recruitment of YTHDF3 and translation initiation factors eIF4G2 and eIF3A. Hundreds of endogenous circRNAs thus possess translation potential (Table 1). Of note, m6A modification is reversely regulated by miRNA. Via sequence pairing with mRNA containing miRNA targeting sites, miRNA modulates METTL3 binding to target RNA, resulting in an increase of m6A modifications. Mouse embryonic fibroblasts are thus reprogrammed into pluripotent stem cells (18).

**Table 1:** m6A affects ncRNA genes.

**m6A targets**	**Writers/effectors**	**Mechanisms /functions**	**References**
pri-miRNA	METTL3/HNRNPA2B1	Recruiting DGCR8 to pri-miRNA transcripts to facilitate miRNA processing	(38, 49)
lnc-XIST	RBM15/RBM15B/ YTHDC1	Responsible for XIST-mediated transcriptional repression	(16)
lnc-MALAT1	HNRNPG	Binding to the m6A-modified purine-rich hairpin to regulate splicing and gene expression	(37)
circRNA	YTHDF3	YTHDF3 recruits eIF4G2 and eIF3A driving translation of circRNA containing m6A modifications	(26)
circRNA	YTHDF2	mediating circRNA stability yet independently accelerates circRNA degradation	(50)

## Physiological and Pathological Immune Functions

m6A modification is necessary in the biogenesis and functions of RNA. Modification aberrancies have been associated with various pathophysiologies. Recently, m6A modification has been recognized as crucial regulator in T cell homeostasis and the immune response to bacterial or viral infection. Selectively altered m6A levels along with other types of immunotherapies may be efficient management strategies in a variety of immunological diseases.

### m6A Methylation and T Cell Homeostasis

**Figure 2:**
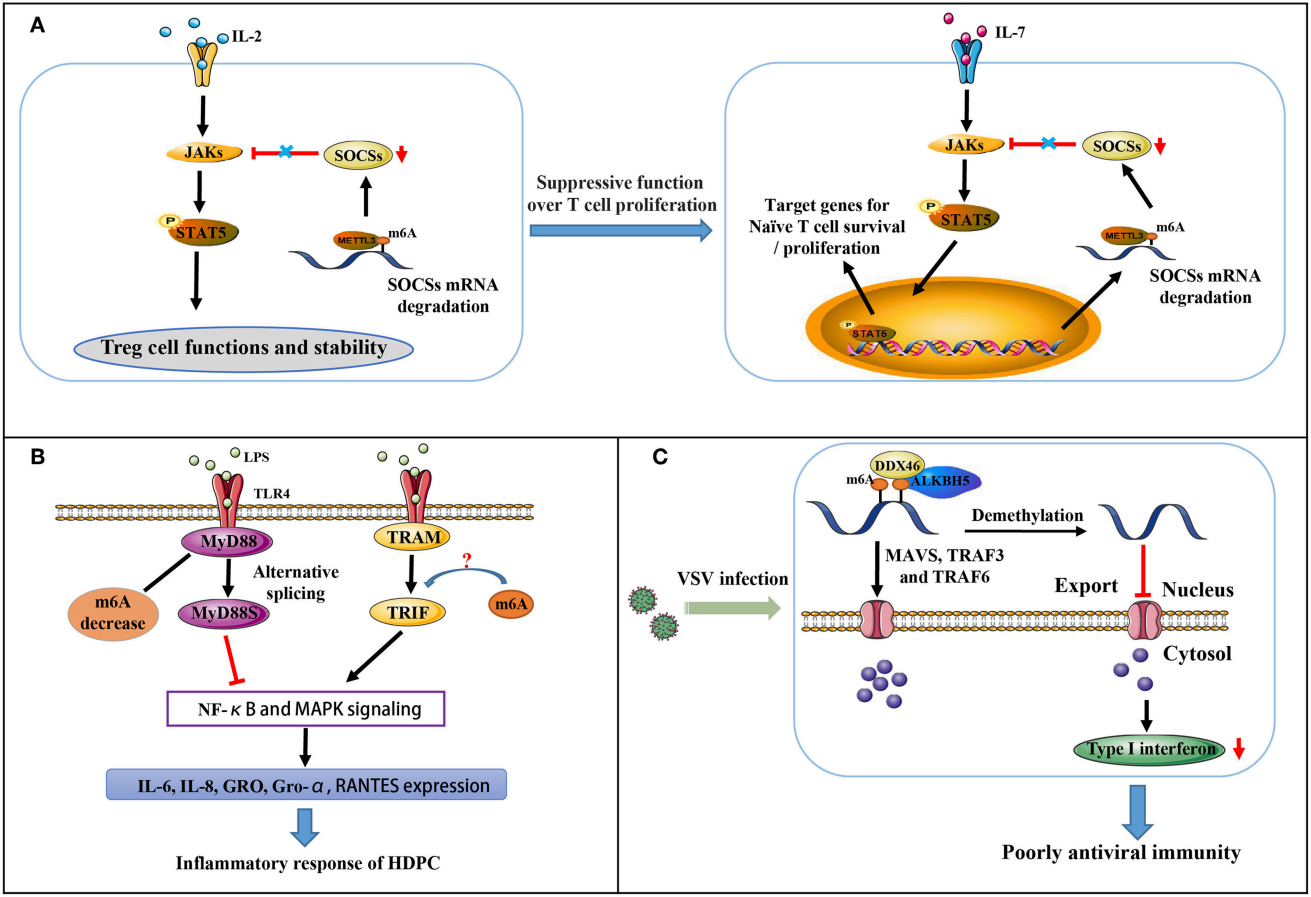
Partial immune mechanisms regulated by m6A modification. **(A)** m6A methylation regulates the suppressive function of Tregs on naïve T cells. **(B)** m6A mediates MyD88 alternative splicing that is responsible for LPS-induced inflammatory reactivity in HDPCs. **(C)** m6A represses type I interferon production in an innate antiviral state.

The suppressor of cytokine signaling (SOCS) protein family encodes inhibitory proteins involved in JAK-STAT signaling, including SOCS1, SOCS3, and CISH, playing a vital role in T cell proliferation and differentiation (51, 52). In naive T cells, which are induced by “gatekeeper” IL-7 stimulation as well-known immediate-early genes, SOCS genes control IL-7 signal and play critical roles in adaptive immunity (51, 52). All three SOCS genes were found to undergo m6A modification via the RNA-IP assay. The modification induces mRNA degradation of SOCS genes that initiates naïve T cells re-programming for proliferation and differentiation. IL-7/JAK signaling is activated *in vitro* and *in vivo* via relieving the inhibition on IL-7-STAT5 signaling (Figure 2A) by an evolutionarily-conserved m6A-dependent mechanism (53). This modification is likely a crucial factor in the regulation of immune homeostasis and the mitigation of various autoimmune diseases.

Regulatory T cells (Tregs) are a crucial specialized T cell lineage, and are involved in reducing inflammation and immunosuppression (54). Chronic intestinal inflammation in METTL3 knockout mice has occurred when the mice reach at least 3 months of age. Co-culture assay of naïve CD4^+^ T cells and Tregs with m6A KO revealed naïve T cells to exert more rapid proliferative influences due to a complete lack of suppressive function for Tregs (Figure 2A) (55). In CD4^+^ T cells, m6A modification is indeed enriched at the GG/AACA/U domain at 3′-UTR and at 5′-UTR of SOCS genes. Decreased m6A modification enhances the mRNA stability of SOCS genes, thereby blocking transduction of cytokine signaling in the IL2-STAT5 pathway (55). As this pathway is critically essential for the suppressive function and stability of Tregs (54), m6A levels are considerably responsible in controlling naïve T cells homeostasis.

### m6A Methylation and Inflammatory Response

Dental pulp inflammation, which can progress to pulp necrosis and periapical diseases, is characterized by a partial accumulation of inflammatory mediators and is a typical inflammatory disease (56, 57). In pulpal and periapical diseases, it is recognized that bacterial infection is a major pathogenic factor (58). Recent findings indicate that in LPS-treated human dental pulp cells (HDPCs), METTL3 expression and m6A modification levels are up-regulated instead of METTL14, FTO, and ALKBH5. Moreover, METTL3 knockdown decreases the expression of LPS-induced inflammatory cytokines, including IL-6, IL-8, GRO, Gro-α and RANTES. At the same time, NF-κB and MAPK signaling pathway activation is suppressed (58). MyD88 exists in two forms (MyD88L and MyD88S). MyD88Land TRIF pathway activate the innate immune response by transducing TLR signals, whereas MyD88S inhibits the response (59). Further research revealed that m6A inhibition significantly increases MyD88S mRNA levels, suggesting that m6A mediates alternative splicing of MyD88 and mediates the LPS-induced inflammatory reaction in HDPCs (Figure 2B) (58). Whether or not m6A also regulates TRIF signaling remains unclear.

### m6A Methylation and Antiviral Immunity

Influenza virus and Rous sarcoma virus were previously reported to produce viral transcripts with m6A modifications. At an antiviral innate state, RNAs containing m6A modifications is unable to stimulate RIG-I-mediated antiviral signaling and induce interferon expression (60). Further research has suggested that m6A modification is involved in the export and translation of signaling molecules, including MAVS, TRAF3, and TRAF6, thus regulating interferon production in the antiviral innate immune response (Figure 2C) (61). The DEAD-box (DDX) helicase family contains 12 conservative domains, many of which have been identified vital in the recognition of viral nucleic acids and regulation of downstream pathways (62–65). DDX46 was recently found to negatively regulate innate antiviral transcripts via recruitment of ALKBH5. This results in reduced m6A levels on MAVS, TRAF3, and TRAF6 RNA and prevents their transport from nucleus into cytoplasm, thus reducing their translation. Moreover, the target mRNA CCGGUU motif is responsible for the effects DDX46 exerts on the antiviral innate immune response by decreasing the production of type I interferons (61). DDX3 also interacts with ALKBH5, the only protein among the identified methyltransferases and demethylases as partnering with DDX3 via the ATP-binding domain of DDX3 and the DSBH domain of ALKBH5. DDX3 is involved in diverse biological processes via the interactions between its different domains and many distinct proteins (66). Interestingly, recent article revealed a seemingly opposing mechanism of m6A in type I interferon response to the herpesvirus human cytomegalovirus (HCMV) infection. m6A level is dramatically upregulated in primary human foreskin fibroblasts infected by HCMV, and required for viral propagation (67). Following infection in METTL3-depleted cells, decrease of m6A modification results in enhanced mRNA stability of IFNB and sustained IFN-β production, the main type I interferon in human non-immune cells, thus triggering a stronger antiviral response to block HCMV growth. Three putative adenosines proximal to stop codon are mechanistically direct targets of m6A, responsible for IFNB mRNA stability. It is probable that for different viruses, the contribution of m6A machinery for immune response may vary even adverse.

Human CD4^+^ T cells infected by HIV-1 can trigger a massive m6A increase in both T cells and HIV-1 mRNA (68). In the CDS and UTR sequences of HIV-1 mRNA, additional splice junctions and splicing regulatory domains have been identified to possess 14 methylation peaks. Gene ontology analysis has revealed that 56 host genes with special m6A modification are active in viral infection. Silencing of METTL3/METTL14 or AKBH5 either decreases or increases HIV-1 replication, respectively. Mechanically, the formation of the HIV-1 Rev-RRE (Rev response element) complex is enhanced by the A7883 site methylation in the stem loop II region of RRE RNA. This complex thereby influences HIV-1 replication and nuclear export (68). Furthermore, one third of the 56 genes which encode diverse functional proteins, including MOGS, TRAF2, and HSPA1A, have been linked with HIV-1 replication. It thus seems that altered host genes modified with m6A are involved in the antiviral T cell immune response due to regulation of RNA expression and biological metabolism.

Kaposi′s sarcoma-associated herpesvirus (KSHV) has been reported as the leading cause of cancer in AIDS patients, resulting in both primary effusion lymphoma and the lymphoproliferative disorders multicentric Castleman′s disease (69). In KSHV-infected renal carcinoma cell line iSLK.219 treated with doxycycline, the level of total m6A was found to be markedly increased, and about one third of KSHV transcripts with m6A modification were found to contribute to KSHV gene expression (70). METTL3 and YTHDF2 are involved in triggering production of virion in cells infected by KSHV. ORF50 protein, a major viral transcriptional trans-activator, upon depletion of METTL3 or YTHDF2 is initially significantly reduced and unable to expedite the expression of KSHV lytic genes. However, ORF50 expression is independent of KSHV infection. m6A initially promotes ORF50 mRNA abundance, but knockdown of YTHDF2 or METTL3 leads to a subsequent negative feedback on the ORF50 promoter. Furthermore, in a KSHV-infected iSLK.BAC16 cell line, ORF50 protein expression is inverted following METTL3 or YTHDF2 depletion, suggesting that m6A variably functions in a cell-specific way. Additionally, m6A regulates newly transcribed viral RNA splicing, stability, and protein translation to control viral lytic gene expression and KSHV replication. Blockade of an event regulated by m6A modification decreases viral protein expression and halts virion production (71). Moreover, m6A also plays important roles in latent and lytic KSHV replication as well as KSHV-induced oncogenesis (72). m6A may be a novel, effective target in the management of KSHV infection.

In 1979, m6A residues were identified on the polyomavirus simian virus 40 (SV40) (73). Overexpression of YTHDF2 substantially enhances SV40 replication in the SV40-permissive cell line BSC40 (74). PA-m6A-seq has revealed that SV40 possesses an early region where two m6A clusters are located, and a late region that possesses 11. Instead of detectably affecting SV40 late transcript alternative splicing, m6A present on VP1 ORF principally increases VP1 expression by expediting translation. By decreasing the formation of SAM without affecting mRNA capping, 3-deazaadenosine (DAA), as an inhibitor of methylation, reduces overall m6A modification (75). Subsequent depletion of m6A via DAA treatment inhibits SV40 replication. Drugs that alter m6A modification levels as needed might be in a position to repress replication of different kinds of pathogenic viruses, in particular of those causing acute infections.

### m6A Methylation and Antitumor Immune Response

Tumor neoantigens are important for generating spontaneous antitumor immunity and for valuating clinical responses to immunotherapies (76, 77). Recent article indicates that m6A-modificated mRNAs encoding lysosomal cathepsins are recognized by YTHDF1 in dendritic cells (DCs), subsequently the binding of YTHDF1 facilitates translation of cathepsins, suppressing the cross-priming ability of DCs. Loss of Ythdf1 inhibits tumors growth and host survival in mice model, owing to elevated infiltration of neoantigen-specific CD8^+^ T cells in tumors. Consistently, DC-specific Ythdf1 depletion enhances the cross-presentation of tumor antigens and the cross-priming of CD8^+^ T cells *in vivo*. Furthermore, given that Ythdf1 depletion promotes IFNγ production, followed by PD-L1 increase in CD8^+^ T cells (78), combining PD-L1 checkpoint inhibitor with Ythdf1 depletion shows stronger therapeutic efficacy. In combination with burgeoning checkpoint blockade strategy, YTHDF1 could be a potential new therapeutic target for immunotherapy.

## Conclusion and Perspectives

Being exquisitely regulated by “writers,” “erasers,” and “readers,” additional repelled proteins or miRNAs, m6A modification relates to nearly any step of mRNA metabolism, as well as ncRNA processing and circRNA translation. There is compelling evidence suggesting that m6A modification is especially critical in a variety of pathologic and physiologic immune responses including T cell homeostasis and differentiation, inflammation, and type I interferon production. Further results have indicated that aberrancies of interferon and Th17 frequencies in systemic lupus erythematosus (SLE) patients may be caused by relevant genes changes for the altered m6A levels (79, 80). Whether functional dysregulations observed in SLE are associated with altered m6A modification warrants future exploration (81). Furthermore, m6A controls stem cell self-renewal, differentiation and pluripotency, additional critical functions in metabolism, as well as metastasis in many cancers (82), including acute myeloid leukemia, glioblastoma, breast cancer and lung carcinoma. Altered m6A modification might thus be an effective therapeutic target in preventing and treating human diseases. m6A exerts a variety of functions that result in alterations of particular functional proteins with m6A on mRNAs.

Though the field of m6A modification has become increasingly attractive, serious challenges in research remain. More advanced technology will be needed for quantification of m6A modification on a transcriptome-wide level and identification of precise m6A sites. How one of the members of writers, readers, and erasers performs its separate functions and interacts with one another remains to be elucidated. It is likely that either FTO or ALKBH5 performs the sole demethylation in different tissues as the presence of one has been found to indicate absence of the other. More biochemical participants in the machinery involved in m6A modification remain to be uncovered, and whether the diverse functions of m6A work in concert or are antagonistic in cellular biological processes remains to be researched in detail. We anticipate that subsequent focus on researching m6A modification in the setting of physiological and pathological processes will enrich our knowledge concerning a variety of conditions, contribute to the advancement of the biological sciences and provide us with novel therapeutic strategies.

## Author Contributions

CZ the first author, contributed to collection of references and manuscript preparation. JF and YZ contributed to the modification of the manuscript.

### Conflict of Interest Statement

The authors declare that the research was conducted in the absence of any commercial or financial relationships that could be construed as a potential conflict of interest.
